# Cardiovascular Disease in the Caribbean: Risk Factor Trends, Care and Outcomes Still Far From Expectations

**DOI:** 10.7759/cureus.52581

**Published:** 2024-01-19

**Authors:** Mandreker Bahall

**Affiliations:** 1 Caribbean Centre for Health Systems Research and Development, Faculty of Medical Sciences, University of West Indies, St Augustine Campus, Couva, TTO

**Keywords:** outcomes, acute myocardial infarction, coronary artery disease, cardiovascular care, cardiovascular risks, epidemiology, cardiovascular disease

## Abstract

Cardiovascular diseases (CVD) are a major public health concern in the Caribbean. Cardiovascular care in the Caribbean revealed encouraging improvements but still less than expectations. This study aims to gain insight into CVD and identify gaps in cardiovascular care in the Caribbean compared to high-income countries. More specifically, this review reports on the epidemiology, CVD risk factors, management practices, and patient outcomes (quality of life (QOL) and mortality). A systematic review of peer-reviewed articles was conducted to assess the CVD of individuals in the Caribbean from 1959 to 2022.Using multiple search engines and keywords, a systematic review of relevant peer-reviewed CVD articles was conducted using the necessary inclusion and exclusion criteria. Relevant data of studies were classified by title, publication year, location, type and size of samples, and results. Further analysis grouped patients by epidemiological profile, CVD risk, management, and selected outcomes (quality of life and inpatient mortality).

From the initial review of 1,553 articles, 36 were analyzed from Trinidad and Tobago (20), Barbados (4), Jamaica (7), along with the Bahamas (2), British Virgin Islands (1), Bonaire (1), and one article from a Caribbean study. The social environment of fast food, sedentary jobs, and stress determinants are postulated to be precursors for an increase in CV risks.

CVD in the Caribbean reveals a high prevalence of CV risks, suboptimal care, poor compliance, and high inpatient mortality compared with high-income countries. Greater efforts are required to improve CVD care at all stages, including in the social environment.

## Introduction and background

Cardiovascular disease (CVD) continues to rise at a fast pace in the Caribbean [1] presenting as myocardial ischaemia, heart failure, arrhythmias, and cardiomyopathies. By far, the commonest condition is coronary artery disease (CAD) which is responsible for the highest death rate [2]. Being the leading cause of death [3], CAD has shown significant interest to all stakeholders since its development and complications are largely preventable. Increasingly, in the last decade, an increasing number of women [4] and young adults [5] are being affected which has stemmed from a change in societal and behavioral forces and influences [6]. Caribbean countries have implemented numerous strategies [7], workshops [8], heads of government and ministerial meetings, and conferences [9] throughout the past two decades. Their effects are yet to be evaluated, and these investments are expected to reduce CVD prevalence, and improve risk management, care, and outcomes. The study aimed to compile the primary findings of CAD across Caribbean studies (epidemiology, CV risks, CAD or CVD, and outcomes such as quality of life (QOL) and inpatient mortality). Specifically, the epidemiologic profile of patients by age, sex, and ethnicity was to be determined, the risks for CAD of various subpopulations and variations with time, management of patients (in terms of timing and medical treatment), and determining evidence-practice gaps in CAD care. This review also identifies research gaps and recommends strategies for the treatment and prevention of CAD that would ultimately help policymakers and other stakeholders make data-driven recommendations to address the epidemic.

## Review

Methods

This systematic review delves into pertinent peer-reviewed CVD literature from five English-speaking Caribbean nations, encompassing Trinidad, Barbados, Jamaica, Bahamas, and the British Virgin Islands with the exclusion of Bonaire (Dutch-speaking) [10]. These included articles on epidemiology, cardiovascular risks, and ischemic heart disease (IHD), along with IHD complications such as heart failure and the quality of life of patients with IHD. Our literature search involved rigorous exploration through various electronic databases, namely Medline, PubMed, Web of Science, Google Scholar, and the World Health Organization (WHO) website, to compile research focused on CVD in the Caribbean. 

**Figure 1:**
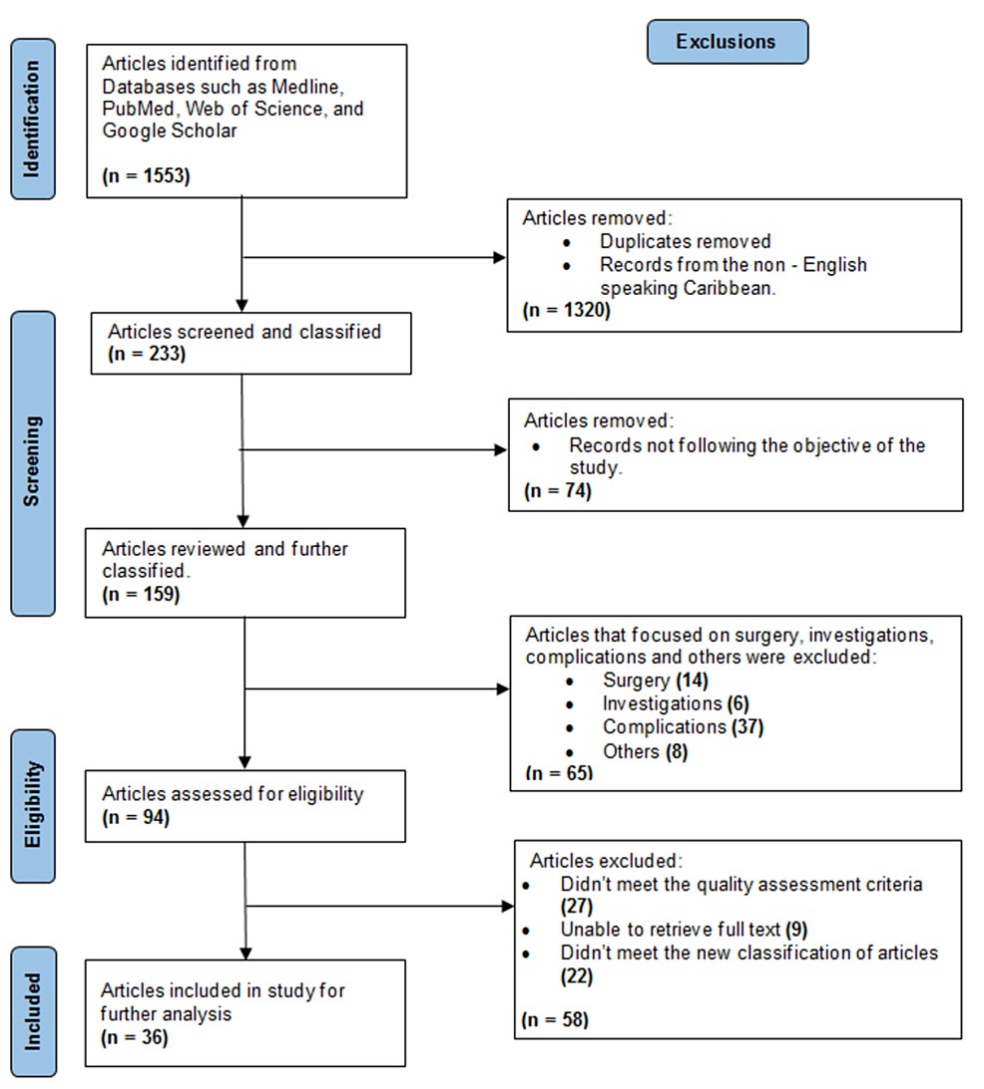
PRISMA 2020 flow diagram for systematic reviews Source: [12] PRISMA: Preferred Reporting Items for Systematic Reviews and Meta-Analyses

The search employed searching for MeSH (Medical Subject Headings) [10] keywords such as acute myocardial infarction (AMI), CAD, HF, arrhythmias, ACS, chest pain, quality of life, cardiac rehab, coronary heart disease, ischemic heart disease, atherosclerosis, risk factors, cardiovascular disease, and names of authors researching CVD in the Caribbean region. The eligibility criteria necessitated peer-reviewed articles from the Caribbean pertaining to coronary health that adhered to specified quality assessment criteria. The study areas encompassed epidemiology, risk factor analysis, nonsurgical treatment, and outcomes of patients with CAD. Articles on surgical interventions, surveillance, review articles, with unclear results, incomplete information, and conference proceedings were excluded (Figure 1). Studies were evaluated using the Quality in Prognostic Studies (QUIPS) tool, while the overall evidence in the meta-analysis was assessed “through the adapted version of the Grading of Recommendations, Assessment, Development, and Evaluations (GRADE) tool for prognostic factor studies” [11]. 

Epidemiology (MOOSE) guidelines for our search strategy**

Data Extraction/Data Analysis/Interpretations

The search yielded articles based on four main themes: epidemiology (four articles) and prevalence (11 articles); cardiovascular risks (11 articles); cardiovascular management: timings (three articles), treatment (three articles), compliance and monitoring (two articles); and outcomes (quality of life and inpatient mortality) consisting of two articles. Data were collected on age, sex, and ethnic distribution of AMI; cardiovascular risks of the population, sub-populations, CAD patients, and AMI patients; management (timing of treatment: pre-hospital delay, door-to-ECG, door-to-needle thrombolytic time); pharmacological treatment (thrombolysis, primary angioplasty, and other medications); and outcome (quality of life and inpatient mortality). Relevant data were entered into a master sheet, classifying the title, year of publication, study location, study sample, objective, summary, findings, and conclusions. Informed consent was waived owing to the retrospective nature of the study.

Results

Of the initial review of 1,553 articles, 36 were accepted for analysis. These studies were mainly conducted in Trinidad and Tobago, Barbados, and Jamaica along with the Bahamas, British Virgin Islands, and Bonaire.

Epidemiology

Alfred et al. (2009) in a Tobago study found that the mean (± standard deviation [SD]) patient age was 58.47±12.66 years in 2007, and 71.00 ± 12.25 years in 2008 [13]. In 2019, “the mean (standard deviation [SD]) patient age was 58.6 ± 13.43 years with the mean age of the women higher than that of the men (62.2 ± 13.30 years vs. 56.9 ± 13.15 years, respectively, p ≤ 0.001)” [14]. In 2016 (South Trinidad), the overall incidence rate of AMI was reportedly 90.6 per 100,000 population [15]. In terms of incidence rates by gender and ethnicity, the highest was in “Indo-Trinidadian men,” of 141 cases per 100,000 population; Indo-Trinidadian women were at 90 cases per 100,000 population; Afro-Trinidadian men at 81 per 100,000 populations; and Afro- Trinidadian women, at 45 per 100,000 populations” [15]. In Barbados, the age-standardized incidence rate (ASIR) for AMI increased from 67 per 100,000 in 2011 to 123 per 100,000 in 2019. Another study from 2011 reported a lower occurrence of AMI, and it is slightly higher among Indo-(n=129, 48.5%) than among African-Trinidadians (n=110, 41.4%) [16]. The AMI was consistently higher in men, ranging from 83 to 120 per 100,000 [17]. The rates in women range from 43 to 83 per 100,000 individuals [17]. 

Cardiovascular risks

*Prevalence of CVD Risk Factors in Caribbean National Populations* 

Hypertension (ranging between 16.6% and 30.2%): Hypertension is prevalent across the studies, varying from 25% in males (M) and 30% females (F) (n=3594) (Bahamas, 1959), 43% (M) and 47% (F) (n=464) (Barbados, 1993), 25% (n=2848) (Jamaica, 2011), 16.6% (n=301) (British Virgin Islands, 2012) and up to 30.2% (n=14,793) (Trinidad and Tobago, 2013) [18-22].

Diabetes (ranging between 7.2% and 19.5%): Diabetes prevalence varies from 17% (n=464) (Barbados, 1993), 13.4% (n=1303) (Jamaica, 1999), 7.2% (n=2012) (Jamaica, 2008), 8% (n=2848) (Jamaica, 2011), 10.0% (n=301) (British Virgin Islands, 2012) and 19.5% (n=14,793) (Trinidad and Tobago, 2013) [19-24]. Impaired glucose tolerance is also prevalent at 12.3% (M) and 14.7% (F) (n=1303) (Jamaica, 1999), 3% (n=2848) (Jamaica, 2011) and 16.9% (n=301) (British Virgin Islands, 2012) [20,21,23]. 

Obesity (ranging between 19.7% and 25%): Obesity is prevalent at 10% (M) and 30% (W) (n= 464) (Barbados, 1993), 19.7% (n=2012) (Jamaica, 2008), 25% (n=2848) (Jamaica, 2011) and 23.6% (n=301) (British Virgin Islands, 2012) [19,24,20-21]. There is a fourfold excess of obesity in women compared to men (n=1303) (Jamaica, 1999) [23]. Overweight rates ranged from a high of 53% (M) and 42% (W) (n=464) (Barbados, 1993) and 46% (n=2012) (Jamaica, 2008) to as low as 27% (n=2848) (Jamaica, 2011) and 25.6% (n=301) (British Virgin Islands, 2012) [19-21,24].

Prehypertension (ranging between 29.9% and 35%): Slight variations in prehypertension are noted but remain similar, from 30% (n=2012) (Jamaica, 2008), 35% (n=2848) (Jamaica, 2011) and 29.9% (n=301) (British Virgin Islands, 2012) [20,21,24].

Smoking (ranging between 15% and 17.8%): Smoking decreased over time, from 17.8% (n=2012) (Jamaica, 2008) to 15% (n=2848) (Jamaica, 2011) to 16.6% (n=301) (British Virgin Islands, 2012) [20,21,24].

Alcohol consumption: Alcohol consumption is moderate at 51.2% (n=301) (British Virgin Islands, 2012) [21].

Hypercholesterolemia: This declined slightly over time, from 14.6% (n=2012) (Jamaica, 2008) to 12% (n=2848) (Jamaica, 2011) [20,24].

A strong inverse curvilinear relation between high-density lipoprotein cholesterol and coronary heart disease incidence was determined (p < 0.005) in West Indian men (n=1246) (Trinidad and Tobago, 1989) [25].

CV risks varied with selected subsets of the population studied, namely, the adolescent population, school population, and antenatal clinic patients. Among adolescents (n=276) in Jamaica (2013), women had more risk factors and were less fit than males (p<0.05). 14.5% of adolescents were overweight, and 21% were obese [26]. Among school children (n=2023) in Bonaire (2013), risk factors included overweight (17%), obesity (12%), and hypertension (13%) of normal-weight children, 23% of overweight children, and 53% of obese children [27]. Among antenatal clinic patients (n=428) in Jamaica (2000), hypertension was negatively associated with placental volume at 17 weeks and fetal abdominal circumference at 20 weeks [28]. Among diabetic patients (n=387) in Trinidad (2000), CVD risk factors included obesity (37%), overweight (35%), hypertension (21%), hypercholesterolemia (25%), and hypertriglyceridemia (22.3%) [29]. Among chronic disease patients (n=734) in Trinidad (2005), depression was prevalent (28.3%) [30]. Among hospitalized patients with cardiac disease (n=388), the prevalence of significant depression was 40% (Trinidad and Tobago, 2019) [31]. 

Prevalence of CVD Risk Factors in Caribbean CAD/AMI Patients

Analyzing data from confirmed AMI/CAD patients, we observed a concerning picture of cardiovascular risk factors:

Hypertension: This is prevalent across the studies, ranging from 19.6% (Trinidad, 2011), 34.5% (Trinidad, 1994), and up to a staggering 75.9% (Caribbean, 2014) [16,32,33].

Diabetes: Diabetes is also prevalent, but with some variation: 13.5% (Trinidad, 2011), 36.4% (Trinidad, 1994) to 47.8% (Caribbean, 2014) [16,32,33].

Smoking: Smoking declined significantly over time, from 70.9% (Trinidad, 1994) to 28.9% (Trinidad, 2011) [16,32].

Alcohol consumption: This is moderate at 32% (Trinidad, 2011) [16].

Obesity: Obesity increased over time, from 12.6% (Trinidad, 2011) to a worrying 48.3% (Trinidad, 2016) [16,34].

Multimorbidity: This is also prevalent, with 43.2% of patients in Trinidad (2011) having both hypertension and diabetes [16]. Overweight rates also climbed, reaching 35.0% in Trinidad (2016) [34].

Cholesterol: High across the region, with 37.8% of Caribbean patients in 2014 having elevated levels [33].

While dietary studies are scarce, a 2023 study in Trinidad provided stark evidence: overwhelming preference for atherogenic foods high in sugar and salt among adults [35].

Management of ACS/AMI

To assess timeliness, the total time taken from symptom detection to hospitalization was measured as the mean pre-hospital delay (PHD) time. In 2018, for patients established with AMI, the mean PHD time varied from between 7.5 and 18 hours [36]. The mean PHD was 7.5±6.6 hours in Trinidad [36]. In the Bahamas, the PHD was 18 hours, where 56% of patients present within 12 hours [37]. A 2017 Trinidad study at a rural emergency department found that “the median door-to-ECG time was 10 min, with 52.5% of patients achieving a door-to-ECG time of less than 10 min. The median door-to-needle time was 70 minutes, with only 8.2% of patients having a door-to-needle time of less than 30 minutes” [38]. In 2019, among AMI patients, 57.5% of patients received thrombolysis within 30 min [14].

A review of treatment patterns in Caribbean CAD/AMI patients reveals that the treatment modality was mainly confined to providing medication. For patients with AMI in the Bahamas, core AMI drugs like oral nitrates (96%), intravenous heparin (90%), and beta-blockers (65%) were prescribed within a range of 65% to 95% [37]. Similarly, studies from Trinidad reported medication use in 77%-87% of patients with AMI, with aspirin (87.1%), clopidogrel (87.2%), beta-blockers (76.5%), ACE inhibitors (72.9%), heparin (80.6%), and simvastatin (82.5%) being the top choices [14]. For cardiac clinic patients, the picture was similar, with medication adherence ranging from 42% to 78%. Common prescriptions included aspirin (77.7%), Vastarel (55.1%), statins (42.9%), and beta-blockers (41.5%) [39]. However, compliance remained a concern, with a 2018 study reporting a rate of only 61.2% [39].

Interestingly, Trinidad (2015) also revealed a high prevalence of complementary and alternative medicine (CAM) use (56.2%) among cardiac clinic patients, with herbal remedies being the most popular choice (85.9%) [40]. A Barbados study echoed these findings, suggesting a similar reliance on CAM alongside conventional medicine. Emergency treatment for AMI patients in Trinidad followed a similar pattern: aspirin (97.2%), clopidogrel (97.2%), heparin (81.3%), and thrombolysis (70.5% for ST-elevation MI patients). However, a concerning gap emerged; none of these patients received primary angioplasty.

Monitoring and compliance, defined as daily consistency in taking prescribed medications, fell short at 61.2% in Trinidad [39]. In Barbados, between 2009 and 2016, only two (aspirin and clopidogrel) of six essential AMI drugs achieved documented prescription rates of 80% or more [41]. A study conducted in Jamaica revealed non-compliance in hypertensive patients (44%), attaining fasting glucose FBG levels of <6.7 mmol/l (24%), and (FBG) levels <8 mmol/l (38%) [42]. Among cardiac clinic attendees in Trinidad, 78.3% (n=270) were non-adherent [43].

Outcomes

Inpatient mortality for AMI patients in Trinidad was 6.18% for men and 7.2% for women [14], while a Bahamas study reported a mortality rate of 19% [37]. Fortunately, for those who survived AMI, their “overall QOL improved over time in all domains: Emotional, Physical, and Social” [44]. A study among stable cardiac disease patients revealed that the prevalence of moderate to severe depression was 34.3% [95% CI (29.6-39.2)] [45].

Discussion

Cardiovascular Risks

**Figure 2:**
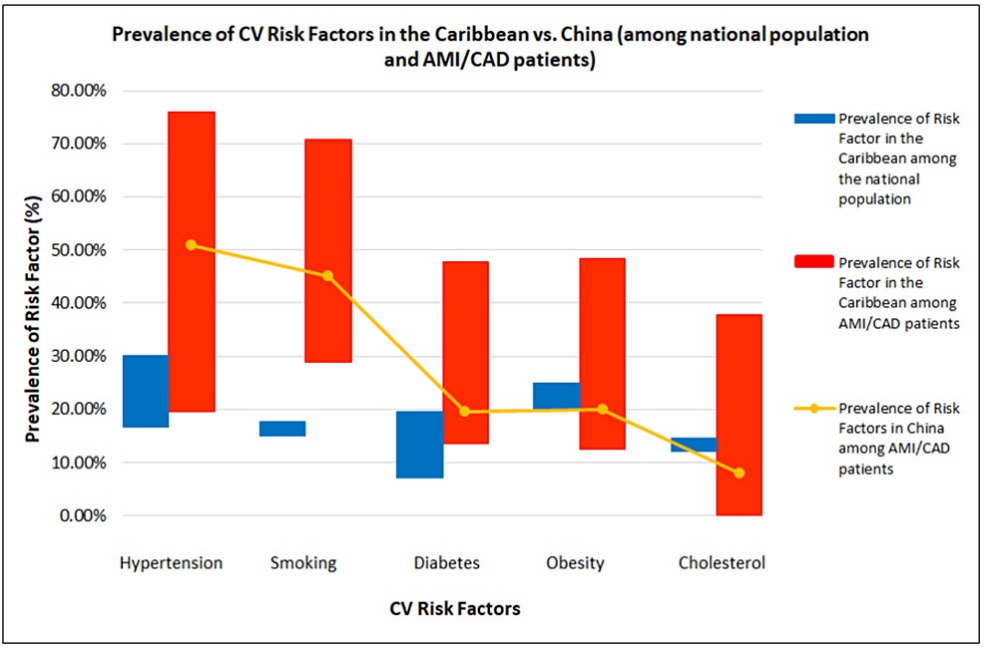
CV risk factors in the Caribbean vs. China among the national population and AMI/CAD patients Source (China): [46-52]

National population studies in the Caribbean reveal the prevalence of hypertension varying between 16.6% and 30.2%, diabetes mellitus (7.2% and 19.5%), and smoking (15% and 17.8%). This contrasts with the much lower prevalence of these risks in high-income countries, such as China (Figure 2). There is an even higher prevalence of CV risk among patients with AMI/CAD in the Caribbean: DM (13.5-47.8%), higher than in China (19.5%) [46]; hypertension (19.6%-75.9%) compared to China’s 50.8% [46]; obesity (12.6%-48.3%) compared to China 20.0% [47]; smoking (28.9%-70.9%) compared to China (45.1%) [46]; cholesterol (37.8%) compared to China (8%) [46] (Figure 2).

**Figure 3:**
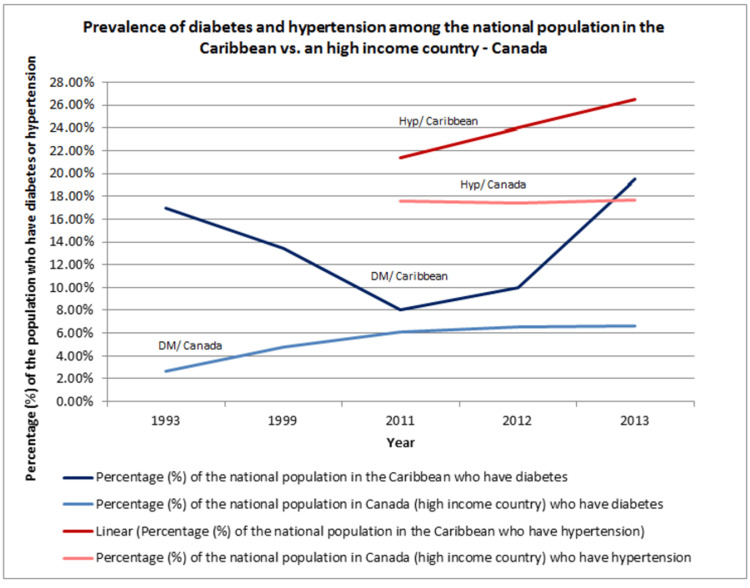
Prevalence of diabetes and hypertension among the national population in the Caribbean vs. a high-income country - Canada (1993-2013) DM: Diabetes mellitus, Hyp: hypertension Source (DM/ Caribbean): [19-23] Source (Hyp/ Caribbean): [20-22] Source (Hyp/ Canada): [53-55] Source (DM/ Canada): [56-60]

The high CV risks may have been fueled by an environment that encourages the development of CAD, such as fast food outlets, inadequate sporting facilities, sedentary lifestyles, and social stressors like murder, family disputes, divorce, child abuse, kidnapping, and rape [6]. Two common modifiable risk factors have shown marked increases over the years, in contrast to those in high-income countries (Figure 3). 

Epidemiology

This increased cardiovascular risk may be responsible for the earlier development of AMI. The incidence of AMI varies between 67 and 123 per 100,000 (Trinidad and Tobago and Barbados), compared to Japan which is 55.2-63.1 per 100,000 [61]. Even when compared to other low-income countries such as Iran (73.3 per 100,000) [62], the prevalence in the Caribbean is significantly higher. The mean age is at least a decade earlier (58.6 ± 13.43 years in 2019) compared to Japan (70 ± 13 years in 2011) [61] and USA (65.6 years for men and 72.0 years for women) [63]. There were more Indo-Trinidadian males than other groups: Indo-Trinidadian males (141 cases per 100,000) compared to Indo-Trinidadian females (90 cases per 100,000); Afro-Trinidadian males (81 per 100,000), and Afro-Trinidadian females (45 per 100 000). 

Management of ACS/AMI

The main focus of treatment was pharmacological which varies between 42% and 97% and compares well with first world countries, varying between 44% and 89% [64-67]. Evidence-based guideline goals for primary angioplasty are virtually non-existent. Furthermore, non-pharmacological treatments such as counseling and cardiac rehabilitation are poor.

Trinidad grapples with a longer pre-hospital delay (7.5 ± 6.6 hours) than that in the United Kingdom (6.1 ± 12.9 hours) [68]. Pre-hospital delay is quite high, with a mean of 1 h more, which is much longer than that in the UK [68]. However, the proportion of patients receiving thrombolysis (door-to-needle time) within 30 min in Trinidad and Canada was similar [69]. The timing of treatment for AMI reveals poor outcomes in low-income countries (Bangladesh) which are far worse than those in high-income countries [70] with the resources available. 

The Caribbean is not alone in its struggles with medication adherence. Similar concerns plague other regions, with a meager 46% compliance rate reported in Saudi Arabia [71]. Adherence to treatment care and goals remain bleak, with less low-income countries attaining HBA1C targets, compared to other countries showing far better outcomes [72]. In the US, non-compliance accounts for around 125,000 deaths and 10% of hospitalizations annually [73,74].

Outcomes

Depression among AMI patients in our study was found to be in 34.3% (n =388 patients) (2018) which is almost twice the reported depression prevalence in the US (18.7%) in 2017 [75]. This disparity is mirrored in in-patient mortality, with our review revealing rates of 6% (males) and 7% (females), while another study reports a significantly higher value of 19% far exceeding the Netherlands' 3% [76]. This may reflect the poor access to quality care for patients in the Caribbean [77]. However, the quality of life of AMI survivors improves with time and may improve with proper intervention, which can be further enhanced through cardiac rehabilitation.

Limitations

Relevant potential studies may have been overlooked due to access barriers. Some abstracts provided inadequate information on their use. Variations in study designs, analyses, and information gathered, and small sample sizes may explain these inconsistencies. Further, the limited time period did not allow for detailed reporting of each study, although there may have been more relevant information.

## Conclusions

This study revealed major deficit gaps in health status in terms of epidemiology, incidence and prevalence, risk factors, treatment timing, optimum management, and outcomes, in the Caribbean. There is a high prevalence of CV risks with the resulting young age of developing CAD which more commonly affects males and Indo-Trinidadians. Over the last few decades, CV diseases have increased across the Caribbean. The growing atherogenic environment and sub-optimization of risk factor management have hastened the earlier development of CAD and its complications. 

We must implement a multipronged approach to bridge these care gaps. This means both robust healthcare systems and broad societal interventions, likely requiring individual efforts as well. The evidence is clear that an action plan is required that may potentially include societal interventions along with individual efforts.
